# Indications, anaesthesia and postoperative protocol for replantation and revascularization in the hand in Nordic countries

**DOI:** 10.1177/17531934221123427

**Published:** 2022-09-27

**Authors:** Henrikki Rönkkö, Ida Neergård Sletten, Karina Liv Hansen, Jorma Ryhänen, Mihai Pietreanu, Jarkko Jokihaara

**Affiliations:** 1 Department of Musculoskeletal Diseases, Tampere University Hospital, Tampere, Finland; 2 Division of Orthopaedic Surgery, Oslo University Hospital, Oslo, Norway; 3 Department of Hand and Microsurgery, Odense University Hospital, Odense, Denmark; 4 Department of Hand Surgery, Helsinki University Hospital and University of Helsinki, Helsinki, Finland; 5 Department of Hand Surgery, Stockholm University Hospital, Stockholm, Sweden; 6 Faculty of Medicine and Health Technology, Tampere University, Tampere, Finland

**Keywords:** Amputation, replantation, revascularization, postoperative care, rehabilitation

## Abstract

In this survey, we compared the current postoperative practices in the largest replantation units of four Nordic countries. The topics included were indication for surgery, anaesthesia, postoperative monitoring, use of antibiotics, anticoagulation and postoperative intravenous fluids, change of dressings, duration of bed rest and hospital stay, hand therapy and follow-up after discharge. Although there were many similarities between the units in the postoperative protocols, we found a large variety of practices. There is no robust evidence to assess or support or reject most of the strategies in postoperative care. The differences in practice warrant prospective studies in order to establish an evidence-based postoperative protocol for replantation surgery.

## Introduction

The Nordic countries have 27 million inhabitants and are socioeconomically relatively homogeneous. All replantation units are located in public hospitals, and all residents in the countries have equal access to emergency medical treatment, including replantation surgery. In Denmark and Norway, replantation surgery is centralized in the cities of Odense and Oslo, respectively, while in Sweden and Finland, it is spread out among several large hand surgery units.

Upper extremity amputations are most commonly cuts caused by power tools, such as circular saws,- and crush or avulsion injuries caused by heavy machinery (Jokihaara et al., 2017). Postoperative treatment is considered crucial for the success of replantation and revascularization of the hand (Allen and Levin, 2002; Prsic and Friedrich, 2019). In the absence of proper evidence-based practice, the objective of this study was to assess the current practice in the four largest Nordic countries to provide a pragmatic view on the indications, anaesthesia and postoperative care for hand replantations.

## Methods

A survey with special focus on postoperative treatment after replantation at the metacarpal or digital level was conducted in the five largest replantation centres in four Nordic countries: Odense University Hospital in Denmark, Oslo University Hospital in Norway, Stockholm University Hospital in Sweden and Helsinki University Hospital and Tampere University Hospital in Finland. Each of these units performs between 20 and 50 replantation surgeries per year. The questions addressed in the survey were postoperative monitoring, use of antibiotics, anticoagulation and postoperative intravenous fluids, change of dressings, duration of bed rest and hospital stay, hand therapy and follow-up after discharge. Moreover, the indication for surgery and the anaesthesia were recorded. Technical details of surgery, data on replant survival and the long-term functional outcome were not addressed.

## Results

### Indications and contraindications

All five centres considered multi-finger amputations or isolated thumb amputations in adults as indications for replantation. In most of the centres, replantation after single finger amputation and amputation distal to the distal interphalangeal joint in adults were not considered. The indications for replantation and revascularization were broader for children, and replantation was generally attempted. There were no age-related contraindications for replantation surgery, but in the elderly the decision was always made individually, taking into account also the patient's concurrent diseases and functional capacity.

### Anaesthesia and postoperative analgesia

In all centres, replantation was performed under continuous brachial plexus block, combined with general anaesthesia in children and, if needed, in lengthy procedures in adults. The plexus anaesthesia was continued until the fourth or fifth postoperative day in Denmark, Finland and Sweden, and in Norway until the ninth day.

### Monitoring

**Figure 1.**
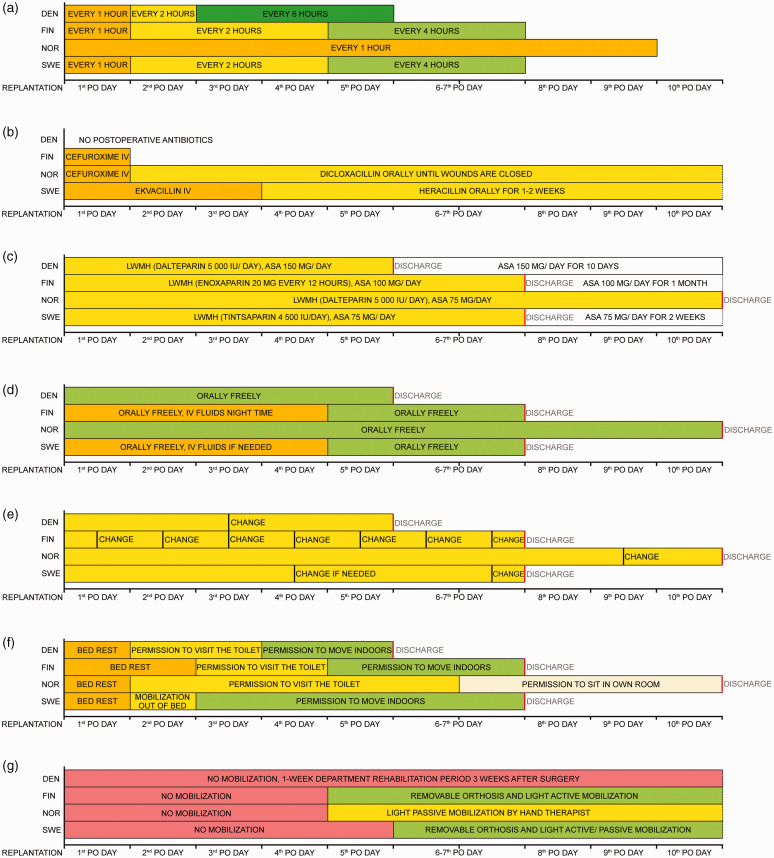
Comparison of practices in Nordic replantation centre. (a) The frequency of vitality checks. (b) The use of postoperative antibiotics. (c) The use of postoperative anticoagulation. (d) Postoperative oral nutrition and intravenous fluid therapy. (e) The frequency of dressing changes. (f) The duration of bed rest and hospital stay and (g) Hand mobilization. DEN: Denmark; FIN: Finland; NOR: Norway; SWE: Sweden; LWMH: low-weight molecular heparin; ASA: acetylsalicylic acid; PO: oral administration.

In all centres, the replants and revascularized parts were evaluated repeatedly by the nurses in the replantation unit. Skin colour, turgor and capillary filling rate were assessed clinically on Likert scales. Temperature was measured with a skin contact thermometer. In all centres, a negative change in the clinical assessment led to evaluation by a hand surgeon for consideration of re-operation or leech therapy. The evaluation was repeated every hour on the first postoperative day in every unit. In all but one centre, the evaluation frequency was reduced to every second hour from the second postoperative day, and to every fourth or sixth hour after 2 or 4 days until discharge. In one centre the evaluation was repeated hourly until discharge (Figure 1(a)).

### Use of antibiotics

Prophylactic intravenous antibiotic treatment was started as soon as possible after the injury and continued until the end of the operation in all centres. After the surgery, there was a variety of practices: antibiotic treatment was stopped immediately after the operation or after the first postoperative day, or intravenous or oral treatment was continued up to 2 weeks or until the wounds were healed (Figure 1(b)). Cefuroxime was the most commonly used antibiotic, but also cloxacillin or flucloxacillin were used. If medicinal leeches were used for postoperative venous insufficiency, fluoroquinolones were administered in all centres.

### Anticoagulation

In most centres, 2500–5000 IU of heparin were administered immediately before vessel clamp opening in cases with crush injuries and increased risk of early thrombosis. One centre used intravenous heparin routinely in all replantation surgeries.

A prophylactic dose of low molecule weight heparin (LMWH) was administered subcutaneously in all centres during the hospital stay. One centre started LMWH administration before surgery, while others started after the primary operation. Seventy-five to 150 mg of acetylsalicylic acid (ASA) was administered daily during the hospital stay in one centre, and for 10 days to 4 weeks in the other three centres (Figure 1(c)).

### Nutrition, fluid therapy, caffeine and nicotine

In most centres, patients were allowed to eat freely right after the operation, but in one centre food was withheld for 12 hours postoperatively. A bladder catheter was used during the operation and removed on the first postoperative day. All patients were encouraged to drink as much as possible. Intravenous fluids were routinely used in one centre up to 4 days postoperatively to complement oral rehydration, mainly during the night (Figure 1(d)). In all centres smoking was prohibited for at least 1 month. Patients were also required to refrain from all other nicotine-containing products for at least 1 month in one unit. Two centres restricted the intake of caffeine during the first 3 or 7 days.

### Dressing change

In all centres, wound dressing consisted of a paraffine gauze or a silicone contact layer, covered by a large and fluffy bandage protected by a cast, avoiding too many gauze folds between fingers as these may compress the anastomosed vessels. The whole upper extremity was covered with a warm blanket and the room temperature was kept constant without cooling air conditioning. In one unit, the blankets were not used because the surgeons felt that it could falsely raise the temperature values. In one centre dressings were changed at least once daily, while in other units the changes were much less frequent or left unchanged until discharge (Figure 1(e)). All centres changed dressings more frequently if there was marked bleeding, as during leech therapy.

### Bed rest, hospital stay, emotional and social care

The patients were confined to bed for the first postoperative day in all centres. In most centres, the mobilization out of bed was restricted to toilet visits during the second and the third postoperative days. The operated hand was always kept slightly elevated. Unrestricted mobilization indoors was gradually allowed on the third through the fifth day. One unit preferred bed rest for up to 10 days until discharge and allowed only sitting in a chair next to the bed for a few hours daily 1 week after the surgery. The duration of hospital stay was typically 5 to 7 days, but routinely 10 to14 days in one centre. During their stay, the patients were offered at least one session with a psychiatric nurse and with a social worker in most centres (Figure 1(f)).

### Hand immobilization and mobilization

In most centres, mobilization and hand therapy was initiated 4 or 5 days after surgery. In one centre, the hand was immobilized with a cast for 3 weeks postoperatively, followed by re-admission to the ward for 1 week of intensive hand therapy (Figure 1(g)). In the centres with an active mobilization scheme, the continuous plexus block anaesthesia was discontinued on the fourth or fifth day, and the cast was replaced with a custom-made removable dorsal orthosis that prevented metacarpophalangeal (MP) joint extension. Hand therapists instructed the patients in light active mobilization: interphalangeal joints were actively flexed and extended while passively keeping MP joints in flexion. One centre had a more conservative approach to mobilization, with gentle passive motion of the fingers controlled by a hand therapist after the first few postoperative days and only careful passive exercises for the following 3 to 4 weeks. In patients who were mobilized after revascularization procedures, the tendons and bones may be intact, and the hand therapy protocols in all centres were more active.

### Outpatient follow-up

At discharge, the patient was in all centres advised to avoid strenuous exercise and stay mainly indoors for the first month after the operation, particularly during the winter. In most centres, the first visit to the outpatient clinic was arranged at 1 to 2 weeks after discharge and included an assessment by both a hand therapist and a hand surgeon. Later visits were planned individually depending on the type of osteosynthesis used and the mobilization scheme, and in most cases the patient met a hand therapist and/or a hand surgeon 1 to 4 times a month depending on the severity of the injury. The initial protective orthosis that allowed active hand mobilization exercises was used for up to 6 weeks. Hand use in daily light activities was usually allowed from 8 weeks, and gradually increasing use of force was allowed after 3 months, while unrestricted heavy weight bearing was usually not allowed until 6 months postoperatively. The first postoperative radiographs were commonly taken at 6 weeks. The fixation material, typically K-wires, were removed when radiological bone healing was ensured, usually at around 8 weeks postoperatively. Plate and screw fixations were infrequently used even in transmetacarpal replantation. Regardless of the frequency of early stage follow-ups, the patients usually had a final visit 1 year postoperatively. In some cases, a longer follow-up was required due to later, secondary surgery. In the early phase, revision amputation of non-surviving tissue and wound healing problems directed surgical treatment. Later, fracture non-union or corrective osteotomies of malunions, or arthrodesis of destroyed and painful joints could be indicated.

## Discussion

We found in this study that although there are many similarities, there was a large variety of practices regarding postoperative care after replantation surgery in four similar neighbour countries.

All the centres performed replantation surgery under continuous plexus anaesthesia. It enables optimal blood pressure for distal tissue perfusion, and it can directly promote peripheral vasodilatation (Bosselmann et al., 2021). The peripheral vasodilatation is particularly important in children, because they seem to have a tendency to develop vasospasm peripheral vessels (Duteille et al., 2003). Postoperative pain can aggravate peripheral vasoconstriction and thus compromise digital perfusion. Continuous regional anaesthesia is also considered as the best postoperative analgesic treatment. The duration of postoperative plexus analgesia differed substantially between the centres in our survey, and we could not find any indication in the literature on the optimal treatment length.

The monitoring practice of the replants was identical in the Nordic centres in this survey, but the length of the observation period varied. We could not find support in the literature for any specific regime. Many methods have been proposed for follow-up of distal circulation, including Clark electrode, pulse oximetry, doppler ultrasound flowmetry and laser doppler flowmetry (Nishijima et al., 2017; Smit et al., 2010), but repeated visual inspection with skin temperature measurement is still the gold standard and universally used procedure. Possible bed-side procedures in case of circulatory problem include elimination of external compression by removing the dressings and some of the skin sutures. If these manoeuvres do not rapidly restore the circulation, immediate return to the operation room is indicated. If re-operation of veins is unfeasible or likely not beneficial because of injury pattern, medicinal leeches can be used to treat distal venous congestion (Hackenberger and Janis, 2019; Pickrell et al., 2020). If clinical signs of circulatory improvement are present after 48 hours of the leech therapy, it is usually continued for 5–12 days during which neovascularization is expected to occur. Other bed-side salvage procedures, such as fish-mount incision in the finger pulp or use of heparin-soaked gauzes after nail plate removal can be used as a last attempt to drain the venous congestion. When the salvage therapy is initiated, further surgical exploration is not considered beneficial.

There were distinct differences in use of antibiotics between the Nordic centres. Antibiotics were always administered preoperatively, but there is no evidence in the literature of the benefits of prolonged prophylaxis. If leech therapy is indicated, fluoroquinolones are used against *Aeromonas* species (Hackenberger and Janis, 2019).

The Nordic schedules for LMWH were diverse, but all centres administered a prophylactic dose to reduce the risk of pulmonary embolism rather than thrombosis in the replant. Other measures to reduce the risk of deep venous thrombosis or pulmonary embolism included passive mobilization of the patient’s legs every hour during the operation and use of compression stockings during the hospital stay. Oral ASA was administered to prevent arterial occlusion. There are several publications on perioperative and postoperative anticoagulant medication (DeFazio et al., 2016; Lee et al., 2016; Nishijima et al., 2019; Reissis et al., 2020; Retrouvey et al., 2019; Zhu et al., 2017). Nevertheless, the benefits of anticoagulant medication during or after replantation surgery remain unclear. The practices of bed rest and hospital stay were quite different in the Nordic centres. Effects of bed rest duration after replantation have to our knowledge not been compared, and the different practices in our units are based on local clinical experience.

In all Nordic centres it was considered beneficial to maintain a neutral or slightly positive fluid balance postoperatively to promote peripheral circulation. The practice differed between the centres in recommendations about caffein and nicotine intake, although regular cigarette smoking has been showed to result in poor success rate (Waikakul et al., 2000). In a retrospective study, Nishijima et al. (2016) found no difference in necrosis rate between smokers and non-smokers treated with prostaglandin E_1_ therapy.

The practice of dressing changes varied greatly. The proposed rationale for daily dressing changes is that moist dressings can cool tissues and dried blood can stiffen dressings and cause local compression. Conversely, others consider that all mechanical disturbance of the replanted tissue should be avoided, and therefore, dressings are changed only if the bandages are constantly saturated or at discharge.

Amputation can cause substantial emotional trauma and financial challenges. In a Finnish study, the median duration of the sick leave after an upper extremity crush injury and distal replantation was 115 days in non-retired adults (Jokihaara et al., 2017). Almost all centres had a routine practice to help the patients with the situation, and all Nordic countries have health and well-fare systems, with sick-leave arrangements and guidance for adjustments or retraining if change of work becomes necessary.

The hand therapy regimes differed, but there is a general agreement that osteosynthesis of the replant must be stable enough to enable early mobilization. Silverman et al. have introduced an early protective motion protocol (Silverman and Gordon, 1996; Silverman et al., 1989). In practice, the activity of individual mobilization programmes depends on many factors, including type of osteosynthesis and tendon repair.

Outpatient follow-up in the Nordic countries was similar. Chronic pain related to nerve injury, lack of tendon excursion and joint stiffness are the main long-term complaints in this patient group. Cold sensitivity caused by nerve injury is probably a more pronounced problem in the Nordic countries due to our cold climate.
